# Commentary: Emotional intelligence impact on half marathon finish times

**DOI:** 10.3389/fpsyg.2018.02593

**Published:** 2018-12-18

**Authors:** Sylvain Laborde, Emma Mosley, Fabrice Dosseville

**Affiliations:** ^1^Department of Performance Psychology, German Sport University Cologne, Cologne, Germany; ^2^UFR STAPS, University of Caen Normandy, Caen, France; ^3^Southampton Solent University, Southampton, United Kingdom

**Keywords:** emotional intelligence, personality, performance, endurance, running

The aim of this commentary is to contextualize the findings of the study “Emotional intelligence impact on half-marathon finish times” (Rubaltelli et al., 2018), which concluded that trait emotional intelligence predicted half-marathon finish times above and beyond training. The aim of this commentary is to highlight some methodological and interpretation limitations that may undermine the conclusions of this paper. These limitations are concerning the acknowledgement of previous research, the choice of the variables included in the study, and the over speculation of findings with respect to study design.

The first recommendation is related to the acknowledgement of previous research findings. The authors do not recognize several pieces of prior work, which would directly inform their research hypotheses and build on pre-existing knowledge within the field. The first paper of interest which was not mentioned is the systematic review of emotional intelligence in the field of sport and exercise (Laborde et al., 2016). The systematic review discussed the theoretical implications of trait emotional intelligence on short-term and long-term sport performance outcomes. It concluded that trait emotional intelligence, given its trait nature, was more likely to have a stronger influence on long-term outcomes in comparison to short-term outcomes. Short-term outcomes may also be impacted, however it is then critical to provide a convincing rationale about why this may be the case, and to choose an appropriate design to test it. The second set of studies that were overlooked originate from sport science and have the exact same aim as the current study, i.e., predicting half-marathon finish times, albeit following a different approach (Knechtle et al., 2014; Gomez-Molina et al., 2017). Knechtle et al. (2014) found that there was a strong correlation between half-marathon finish time and anthropometric and training variables (*r* = 0.89). In a similar vein, Gomez-Molina et al. (2017) showed that half-marathon finish times could be very accurately predicted (to more than 90%) when taking into account (objective) training, anthropometric, physiological, and biomechanical variables. While these two studies did not integrate any psychological variables, it seems reasonable to conclude that the objective variables they assessed should not be neglected when attempting to predict half marathon finish times. Although the authors do acknowledge the importance of physiological factors in their limitations, they do not cite the evidence that directly supports the need for physiological investigation (Knechtle et al., 2014; Gomez-Molina et al., 2017). Finally, the short-form of the trait emotional intelligence questionnaire (TEIQue-SF) was used in this study. Again directly relevant literature was not highlighted, as the short-form has been found to give systematically higher scores than the full-form of this questionnaire (Laborde et al., 2017), which may have impacted the correlational findings of this study.

The second limitation is the choice of variables included in the study, beyond the absence of any (objective) training, anthropometric, physiological, or biomechanics variables already mentioned above. The introduction of the paper justifies the potential influence of trait emotional intelligence on half-marathon finish times based on various mechanisms suggested as mediators, broadly spanning from fatigue, motivation, emotional states, or optimism. However, these variables were not assessed in the study, which limits the ability to draw sounds conclusions about the mechanisms involved. This methodological drawback leads to the third limitation we identified, the over speculation made by the authors about the findings.

The third limitation is linked to the interpretation of the findings that do not match the design and statistical analysis adopted in this study. On the one hand this relates to the methodological consideration detailed above, i.e., not including key predictors variables in the study. On the other hand there is a misconception of elaborating causal explanation from structural equation modeling results. With regard to methodological consideration, an example would be the speculation that performance is related to the management of negative emotions induced by fatigue. Given neither fatigue nor negative emotions were measured in this study, we do not know if negative emotions did lead to poorer performance during the half-marathon. On the contrary, there is a large body of research showing that emotional valence (positive vs. negative) is less important in the prediction of performance than emotional functionality (functional vs. dysfunctional) (Hanin, 2007). Following this line, previous research found that some runners intentionally increase negative emotions such as anger before a race with the purpose to increase performance (Lane et al., 2011). With regard to the findings of this study being based on structural equation modeling, the results are presented as if a causal relationship would exist between the variables, while the design of the study is purely correlational in nature. A closer look at the correlation table shows for example that in this sample low trait emotional intelligence is associated with a lower number of hours of training. This would then potentially explain the relationship found between trait emotional intelligence and performance (*r* = 0.78), a surprisingly high correlation in comparison to the correlations usually observed between psychological individual differences variables and sport outcomes, which definitely warrants replication in other samples. Taken together, those examples show that the design and statistical analysis chosen make it difficult to understand the variables predicting half-marathon finish times.

In summary, the purpose of this commentary was to contextualize the findings of this study, in order to guide future research. Personality and individual differences can account for a certain range of variation in sport performance and are an important area of research. However this should not cloud the bigger picture of factors influencing physical performance, and if time or funding constraints do not allow researchers to capture all these factors, the important thing is to thoroughly acknowledge them in light of study findings. In this case it would lead runners and researchers reading the conclusions of this study to focus on developing one's emotional intelligence over spending time training, while if we consider the bigger picture and take into account research from different fields (Knechtle et al., 2014; Gomez-Molina et al., 2017), improving half marathon finish time would be a far more holistic affair.

## Author Contributions

SL wrote the first draft of the manuscript. EM and FD provided critical comments to improve it.

### Conflict of Interest Statement

The authors declare that the research was conducted in the absence of any commercial or financial relationships that could be construed as a potential conflict of interest.
